# Positive Psychology 2.0 in a Foreign Language Classroom: Students’ Emotional Experience in English Classroom Interaction in China

**DOI:** 10.3389/fpsyg.2021.789579

**Published:** 2021-12-02

**Authors:** Yingna Wang, Mateusz Marecki

**Affiliations:** College of Foreign Languages, Jilin University, Changchun, China

**Keywords:** positive psychology, SLA, emotions, classroom interaction, EFL classrooms

## Abstract

The second wave of positive psychology (PP 2.0) focuses on the way positive and negative psychology complement each other in social contexts. It offers a balanced interactive model that aims at enhancing the optimal learning outcome through the interplay of positive and negative emotions. Building on a large qualitative study of students’ and teachers’ experiences in EFL classrooms in China, this paper argues that adopting the principles of PP 2.0 could deepen our understanding of learners’ emotional experience in SLA. Using one illustrative case, it shows the dynamic and complexity of students’ shifting emotions as they interact in the classroom over a span of 2 months. One major finding is that the students’ positive emotions could transcend negative emotions and influence their engagement in classroom interaction. This study contributes to the existing research into emotional experiences of classroom interaction that integrates the observable, reflective, and participatory. It draws on interrelated sets of data, including a student and teacher profile questionnaire, classroom observation and recording, student and teacher reflective journals documenting their classroom interaction experiences, and stimulated recall interviews based on recordings and reflective journals. The study in the first place has implications for English teachers and teacher trainers in China and abroad as well as researchers interested in the role of emotional experience in English language learning and teaching.

## Introduction

Language learning is an emotionally charged experience. Until the 21st century, the field of second language acquisition was dominated by studies and narratives focusing on learners’ negative emotions, such as learning anxiety (e.g., Botes et al., 2020). However, with a growing interest in positive psychology (PP), Second language acquisition (SLA) scholars like Jean-Marc Dewaele and MacIntyre (2014) have attempted to address that epistemic imbalance by shifting their attention to positive factors that enhance the learning experience and enjoyment, including well-being and happiness. Still, their shift toward positive emotions brings into question the role of negative emotions in the research process. It has been pointed out that PP tends to ignore the reality and benefits of negative emotions and experiences. Thus, this paper seeks to promote a more balanced model of PP 2.0 to stress the importance of exploring how positive and negative emotions interact and to compensate for the lack of such studies in learning contexts, specifically in the English as a foreign language (EFL) classroom context in China.

Extensive survey studies and structured interviews could, to some extent, offer a comprehensive and more nuanced picture of students’ emotions in foreign language classrooms. However, such methods may fail to recognize the dynamics of learners’ emotional experience in the immediate context of interaction. It is thus worthwhile to adopt multiple perspectives and explore immediate learning environments to better understand students’ emotions in classroom interaction, which is also the focus of this paper.

The emotional experience of Chinese learners may not necessarily be the same as that of learners in a different cultural context (Jiang and Dewaele, 2019). In the prevailing Chinese learning model, which builds on Confucian philosophy, students highly respect their teachers. They also rely on their educators to allocate turns before students present their ideas. This traditional teacher-centered approach provides a chance for learners to reflect before disturbing others in large classrooms (Jin and Cortazzi, 2006). It was influential in English teaching in China for 200 years until communicative language teaching was introduced in the 1980s.

Although communicative language teaching (CLT) was advocated in China when it emerged, its feasibility and practical value have been questioned. Its limited success led to a re-employment of the traditional approach. Thus, some researchers later proposed an eclectic model that combines the two approaches. However, four decades have passed, and the traditional approach remains prevalent in English language classrooms in China today. It shows that educational reforms inevitably affect teachers, their teaching practice, and consequently, students’ experience in interaction. However, the issue of students’ emotional experience in interaction remains largely unexplored under the dual influence of the teacher-oriented approach and CLT.

In this paper, interaction refers to acts of verbal communication, including observable social exchanges between students and the teacher as well as private, unobservable interactions. Informed by sociocultural theory (Lantolf, 2000), it could be considered a communicative activity and a cognitive, social, and cultural activity in a particular context. Previous studies suggest that L2 learners’ willingness to communicate (WTC) in EFL is closely related to a host of individual and contextual factors (Peng and Woodrow, 2010; Ghonsooly et al., 2012). Emotions, especially positive emotions like enjoyment, play a crucial role in enhancing the WTC (Dewaele et al., 2018).

A glance at the research on SLA in the Chinese context reveals that the question of students’ emotional experience of classroom interaction has been largely underexplored. Clearly, there is a need for a holistic perspective to investigate how diverse emotions interact in learning contexts. Therefore, inspired by principles of PP2.0, this paper rests on the belief that learners’ emotional experience, both positive and negative, and anything in-between in classroom interactions, need to be further explored within the context of interaction to gain a better understanding of the process of EFL classroom language learning. Here the context includes both the immediate context of interaction and the broader educational and sociocultural context. Students’ emotional responses before and after interactive classroom activities may be different. This study seeks to deepen our understanding of students’ emotional experiences in EFL classroom interaction. The findings could provide implications for researchers and practitioners within the specific Chinese context and, more generally, for English teachers, teacher trainers, and researchers whose work is focused on understanding interaction in English language learning and teaching.

## Literature Review

### The Second Wave of Positive Psychology

Positive psychology is the scientific study of what goes *right* in life (Peterson, 2006) and what contributes to personal growth. The term was first proposed by Maslow (1954), whose work revolves around the positive aspects of human nature, such as fulfillment and self-realization. Its modern rendition originated from Seligman’s presidential address of the American Psychological Association in 1998. Seligman and Csikszentmihalyi (2000) argued for the importance of creating a new branch of psychology concerned with the positive aspects of human experience. Dubbed positive psychology, it is based on three pillars: positive experiences, positive character strengths, and positive institutions. The former two have received significant attention: studies on positive experiences, especially positive emotions, have generated new knowledge. In terms of character strengths, a large-scale survey was used to research 24 strengths with seven million respondents (VIA Institute, 2019). On the other hand, the third pillar of positive psychology, the positive institutions, has attracted less attention to date. However, it would be worthwhile to explore the ways of setting up positive classrooms to facilitate language learning.

Some prominent theories within the field of positive psychology include the broaden-and-build theory and control-value theory. The former argues that positive emotions are vital for optimal functioning and well-being. The effects of positive emotions include broadening peoples’ short-term thought-action repertoires, building one’s long-lasting personal resources, and reducing undesirable effects of negative emotions (Fredrickson, 2013). The control-value theory of achievement emotion addresses the interactive effect of control and value on emotions (Pekrun, 2006). Achievement emotions are emotions that are linked to achievement activities or outcomes. When a student is interested in the learning tasks and believes they can perform them well, they will enjoy the learning process. However, if the student does not value the task or fails to master the learning objective, they will dislike the learning activity (Putwain et al., 2018).

The rapid growth of positive psychology has also raised concerns about neglecting the significance of negative experiences. Researchers maintain that positive psychology is an addition to mainstream psychology. However, the relationship between the positives and negatives remains debatable. To address this issue, Wong (2011) drew upon Viktor Frankl’s theory of self-transcendence and further extended the scope of positive psychology to lay the foundations for what is recognized as the second wave of positive psychology (PP 2.0), or existential positive psychology. This relatively new branch of study is defined as “the scientific study of virtue, meaning, resilience, and well-being as well as evidence-based applications to improve the lives of individuals and society in the totality of life” (Wong, 2011, p. 72). Its principles include accepting and embracing negative emotions with courage. The quest for self-transcendence motivates us to sustain well-being and overcome difficulties. PP 2.0 stresses the importance of building up positive emotions and confronting or transforming negative emotions to balance the two, providing a credible account of the limitations of positive experiences and the benefit of negative experiences. PP2.0 is more context-sensitive and complex, introducing social and cultural factors to the equation (MacIntyre, 2016).

### Positive Psychology in SLA

Positive psychology is concerned with the well-being of individuals in communities. Well-being is also vital for language learners, so exploring the relationship between positive psychology and SLA would be valuable. However, it was not until the last two decades that an essential component of positive psychology—positive emotions—was sidelined and marginalized in SLA. Previous research on emotions in SLA primarily focused on negative emotions, mainly on foreign language anxiety (Dewaele and MacIntyre, 2014). However, the results were inconsistent regarding whether anxiety influenced students’ L2 performance positively or negatively. The unbalanced focus on negative emotions resulted in a broad spectrum of emotions in L2 classrooms being unnoticed.

Influenced by the development of PP, MacIntyre and Gregersen (2012) were among the first scholars to explore PP in SLA. Building on the broad-and-build theory, they argue that teachers can influence students’ emotions by guiding students to harness the power of positive emotions through imagination. In 2014, the first conference on the Psychology of Language learning (PLL) was held at the University of Graz, and its main aim was to consolidate this emerging field of research. A special issue on PP in SLA, co-edited by MacIntyre and Mercer (2014), points out the advantages of PP in SLA over mainstream psychology, arguing that the former is open to a wide range of epistemological and methodological stances.

Since 2016, PP in SLA has moved from the periphery to the mainstream (Dewaele et al., 2019), as marked by the founding of the International Association for the Psychology of Language Learning in 2018. MacIntyre et al. (2016) edited a book focused on PP in SLA. Researchers have studied foreign language enjoyment as affected by students’ gender, age, language proficiency, attitudes toward foreign language. Other teacher-dependent factors seem to be more related to students’ FLE than to their FLA (also see Wang Y. et al., 2021).

In the past two decades, a positive psychology perspective in SLA has produced a large body of knowledge. Its principles echo a social turn in SLA (Block, 2003), whereby language learning is described as a joint, social, and interactive endeavor. Individual differences and experiences are vital in understanding the process of language learning. With the recent boost of work on PP in SLA, researchers recognized an emotional turn in applied linguistics, which complements the affective turn (White, 2018).

One of the most influential contributions of positive psychology in SLA is the distinction between positive and negative emotions (MacIntyre, 2016). Researchers believe that negative emotions may cause an individual to be more focused on a specific action. For example, anxiety may lead to avoidance. On the contrary, based on the broaden-and-build theory, positive emotions may cause a second language learner to have a broader vision and foster their engagement in the language classroom, building resources for learners’ future interaction and preventing negative emotions in language learning. However, its undeniable merits aside, PP tends to separate and polarize positive and negative emotions.

Primarily influenced by the second wave of positive psychology, researchers in SLA have drawn more attention to the relationship between positive and negative emotions (Dewaele and MacIntyre, 2014). They have also found that (1) foreign language anxiety and enjoyment are negatively correlated and that (2) enjoyment and anxiety are two separate but interrelated dimensions of experience: they can be both high or low, which contradicts the common sense of a seesaw relationship. Li et al. (2020) used questionnaires and open questions to analyze the relationship between foreign language classroom anxiety (FLCA) and foreign language enjoyment (FLE) and EFL achievement. They found a negative correlation between FLCA and FLE. Foreign language enjoyment is negatively related to self-rated proficiency.

In terms of methods, most previous studies used survey studies to analyze positive emotions quantitatively (Boudreau et al., 2018). Some scholars adopt the repeated study of small samples in classrooms (MacIntyre et al., 2019) or controlled laboratories (Boudreau et al., 2018). However, positive emotions have not been fully theorized. We still need to delve into how positive and negative emotions interact and what role they play in facilitating language learning. Studying this interplay would enrich our knowledge of how emotions influence language learning and teaching practice. Other SLA methods may help explore the interplay between positive and negative emotions in language learning. For example, there is a gap in the literature regarding the adoption of triangulation in understanding learners’ emotions, which encompasses some major qualitative methods, such as microanalysis of emotions in the classroom or multiple interviews on different time scales. In addition, there is a need to include multiple perspectives to gain a more comprehensive view of students’ emotional experiences.

### Studies on Positive Emotional Experiences in EFL *C*lassrooms in China

Research on students’ emotional experience in EFL classrooms, especially that clustering around positive emotions, is still scarce. Most adopted survey studies focus on the two most commonly explored factors: foreign language enjoyment (FLE) and foreign language classroom anxiety (FLCA). It was not until recently that some other emotions were explored. Take, for instance, recent research on the boredom of Chinese university students in English classrooms from the perspective of the control-value theory (Li, 2021). In a study of 320 EFL students, it has been indicated that the students’ FLE levels were influenced by three factors: enjoyment of teacher support, student support, and foreign language learning (Jin and Zhang, 2021). Another study employs a Chinese version of the foreign language enjoyment scale to identify three dimensions of FLE related to the teacher, personal progress, and atmosphere, with the first one being at the highest level (Li et al., 2018). It suggests that a variety of internal and external factors influence an individual student’s experience of FLE. Among those factors, the role of the cultural factor in shaping students’ emotions in the Chinese context has been investigated. For example, Jiang and Dewaele (2019) demonstrated that Chinese students’ FLE levels are similar to other students but have a higher foreign language classroom anxiety level than the international sample. It shows that students’ level of FLCA is related to students’ internal variables, while their FLE is linked to teacher-related variables. Li et al. (2021), on their part, studied the combined effect of person-environment factors in shaping emotions. They interviewed 1,718 middle school students and 1,295 college students and found that trait emotional intelligence and classroom environment can predict FLE and FLA. FLE was affected more by the classroom environment compared to that of trait emotional intelligence. However, FLA was less influenced by the classroom environment and more so by trait emotional intelligence. In another study, Wang H. et al. (2021) extend the scope beyond FLE and FLCA by investigating more academic emotions, including enjoyment, pride, anxiety, and boredom. They point out that the class social climate and language mindset influence both positive and negative emotions, which in turn affects learners’ willingness to communicate in and outside class.

Besides the survey studies, there have been studies that use a mixed-method by supplementing some qualitative data. Fang and Tang (2021) studied the relationship between Chinese English learners’ learning anxiety and classroom enjoyment by adopting a positive psychology perspective. Their survey of 140 students and interviews with six students showed no significant correlation between learners’ FLE and FLCA. Peer experience is the most frequently mentioned source of FLC, and fear of being judged negatively is the most frequently mentioned source of FLCA. In their large survey study, which also included one open question, Li et al. (2021) investigated the possible causes for the relationship between emotions and learning achievements. However, as recognized by the authors, a retrospective questionnaire could not capture the short-lived and dynamic features of classroom emotions. Although some qualitative narrative could show some features, it is rather limited.

As discussed above, there is still a gap in understanding participants’ experience in second language learning holistically in the Chinese context. In particular, a more detailed description of how individual learners’ emotions change and interact in a short or a more extended period is worth exploring. Thus, this study uses triangulations and combines different data sets in EFL classrooms in China (including multiple interviews over different time scales) to explore how students experience classroom interaction emotionally from students’ and teachers’ perspectives.

## Methodology

### Research Question

In bridging the gap identified in the literature review above, this paper aims to address the following research question: how do students experience classroom interaction emotionally in EFL classrooms in China? Drawing upon the principles of PP 2.0, this study considers both positive and negative emotions and explicitly focuses on the interaction between the two.

### Participants and Setting

It builds on data from a second-year intensive reading course for English majors trained to be future English teachers at a university in China. The 23 Chinese students included in the study were about 18 years old at the time. Mandarin was their first language, and they had studied English for 9.5 years on average, ranging from 6 to 12 years. Most of them were female students (only two were male), typical for such English major programs in China. Most of them had passed the TEM-4 test, which is equivalent to CEFR B1^+^ to B2 in language proficiency level (Liu, 2012). They came from diverse educational, personal, and social backgrounds and had different values and beliefs. Based on the profile data, the students were from eight different provinces and regions in China. They have reported being particularly confident with their reading and writing skills and less so with their listening and speaking skills. Although not all students chose the English major out of their interest, they believed that enrolling in this program could lift their employment prospects in China.

The teacher of this compulsory course is a Chinese man in his thirties. He holds a Ph.D. and has 6 years of teaching experience. He has once worked as a teaching assistant at an American university and has been awarded a prize for excellence in teaching. He summarized his teaching style as “American,” which differed from the style preferred by what he called “ancient teachers.” By characterizing himself in that way, he wished to strictly distance himself from the traditional teacher-led teaching model and consciously promoted more student-interaction interaction in his classroom, which may also link to his previous working experience.

The intensive reading course was chosen because it takes the most credits required by the national English teaching syllabus in China. As a compulsory course, it aims to develop students’ overall English proficiency, not being limited to reading comprehension. Students are required to take this class in four modules over their first two academic years. They attend it three times a week, each lasting 90 min. The coursebook is *Intensive Reading for Contemporary College English*, Book Four, in which Text A is the main focus in each unit. The final exam is based on textbook content, so the teacher follows the textbook closely. At the same time, the course instructor tries to organize related activities to encourage students’ involvement in classroom interactions.

### Data Collection

In order to have a more comprehensive picture of participants’ experiences in classroom interaction, the ongoing data collection was guided by the grounded theory approach, following the “all is data” principle (Glaser, 1998, p. 145). It allows gleaning as much information as possible related to participants’ experiences. It also allows the data collection and analysis to be driven primarily by participants’ emic perspectives. Four interrelated and mutually informing data sets were elicited.

**FIGURE 1:**
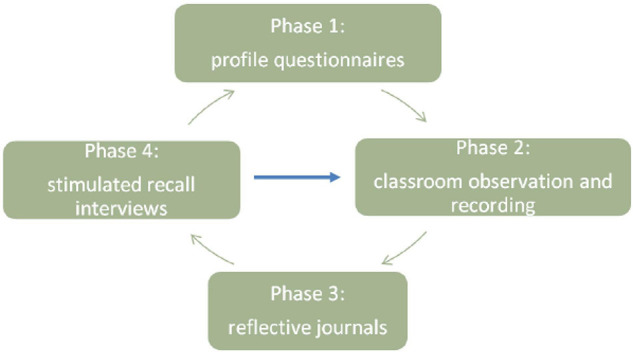
The data collection process.

The data collection process can be divided into four phases (Figure 1). Phase One involves collecting students’ and teachers’ profile questionnaires. Their background information, including their past experiences of second language learning and teaching, were collected, providing valuable information for understanding the social, cultural and prior experiences participants brought to the interaction. As stated in the introduction, interaction is understood as a cognitive and sociocultural activity in this study. Participants were viewed in terms of their past learning experiences, and specific sociocultural profiles, and ways to understand the English language learning process. Thus, a study exploring participants’ experience in interaction needs to include their profile data.

In Phase Two, the researcher observed and audio recorded classroom interaction while taking field notes. As explained in the introduction, this paper is based on a larger study exploring participants’ interaction experience, following the grounded theory approach, and the researcher was an unmotivated observer. The field notes were mainly used to locate the segment of recordings highlighted in students’ reflective journals and used as stimulus material in Phase Four. Therefore, the researchers’ priming effect of prior knowledge of influencing the results of observation was avoided.

In Phase Three, the students and the teacher wrote reflective journals and sent them to the researcher on the same day. They were asked to identify two or three “interactions of particular importance” from that particular session. Then, the researcher identified the episode of interaction mentioned in the journal.

In Phase Four, stimulated recall interviews were conducted the following day. The researcher played the recording identified in Phase Three and asked open-ended questions. The last three phases took place concurrently. During this process, priority was given to participants’ experience, which drove the following data collection. To be more specific, priority was given to what participants said and thought. For example, students’ reflective journal was first analyzed after observation to identify and locate corresponding classroom recordings later used in stimulated recall interviews.

The whole data collection cycle (phases 2–4) was run three times over a month. The researcher’s understanding reached a more profound level in this iterative process. To minimize the risk of participants growing exhausted and affecting their responses’ validity, their reflective journals were sent to the researcher *via* email once a week on the same day after the observation. Then a stimulated recall interview was scheduled one day after the observation to allow enough time for rest.

### Data Analysis

In order to collate and analyze the four data sets, the researcher used a software program called NVivo. This computer-facilitated qualitative analytical tool ensured that data around each identified interaction could be retrieved. While preparing to import the data into NVivo, the researcher also transcribed classroom recordings and interviews. The preparation of data also involved translating the Chinese passages in the transcript and journals into English. When importing the four data sets to NVivo, students’ and teachers’ names were removed and replaced with numbers and pseudonyms.

In order to integrate the four data sets, the researcher developed a construct of “interactional events.” This concept has been developed based on the sociocultural theory (SCT) of interaction, Erving Goffman’s work on the primary framework and the authors’ prior and emerging understanding resulting from data analysis. Informed by SCT, interaction is highlighted in mediation through culturally constructed artifacts (Lantolf and Thorne, 2006, p. 73). Goffman (1974) argued that participants bring their past experiences to interaction. He called this process the primary framework. An interactional event brings together four aspects relevant to the study focus: participants’ background information, the context of interaction, observable interaction, and participants’ experience of interaction. The interactional event acted both as a theoretical and analytical category. Only by taking interaction as a whole, as an interactional event, could we integrate the four data sets to yield insights into interaction and experience of interaction.

Interactional event is an analytical tool that allows studying one instance of interaction from multiple perspectives while considering immediate and sociocultural contexts. In each interactional event, the observational data provides insights into how the interaction was accomplished. The unobservable reflective data can reveal how participants feel and what they think. Combining these aspects brings together their past experiences to the immediate context, which in turn offers insights into the complexities of experiences in interaction.

The process of identifying one interactional event started with an analysis of the journal entries and the creation of a node in NVivo. Then, relevant information was first identified from the students’ journals and gathered under this node from the students’ and teachers’ journals, interviews, and corresponding classroom transcripts. The whole project concluded with the identification of 92 interactional events. When ordered, the number of sources of files was gathered around each interactional event. The most representative case, which ranked number one with 18 files, was “involve me, I learn.” Each data set provided a different lens for understanding the participants’ experience. NVivo software makes it possible to retrieve the original data, compare different perspectives, and maintain sensitivity to the context of specific events.

While coding the data, the study followed a thematic analysis pattern and a constructivist grounded theory approach through initial coding, focused coding, and theoretical coding (Charmaz, 2014). Theoretical coding in this study does not aim to propose a theory but rather to understand participants’ experience in interaction in an EFL context. As Charmaz (2011) argues, researchers with different research aims can adopt grounded theory guidelines in qualitative analyses without necessarily aiming for theory construction. The major theme—emotional experience—emerged as an integral aspect of participants’ experience in each interactional event.

While processing the gathered material, the researcher repeatedly immersed and interacted with the data throughout the data collection, transcribing and reading the data back and forth and keeping memos. The aim at that stage was to identify the most prevalent themes that would bring us closer to understanding the participants’ experience. While coding the data across interactional events, the integration of the four data sets was maintained in generating codes shared across events by these participants. The coding process was led by the reflective data, confirmed by the observational data, and supplemented by the profile data.

## Results

### Context of the Interactional Event

In this section, one interactional event— “involve me, I learn”— is presented as an illustrative case. First, the context of the interactional event serves as a background for participants’ experiences. Second, classroom interaction transcripts are provided to show participants’ experiences from the perspective of observers. Then, reflective data shows participants’ emotional experiences from both students’ and teachers’ perspectives.

The interactional event “involve me, I learn” focuses on one student called Meng and her teacher as they discuss an article entitled *Soldier’s hear*t in the textbook. During this interaction, Meng’s and other students’ emotional experiences were also revealed in the data. When this interaction happened, the researcher observed this class three times, collected two rounds of reflective journals, and conducted two rounds of stimulated recall interviews with the teacher and students. Based on the profile data, Meng passed the TEM-4 (62), which was below the class’s average score (66). She explained that she chose to major in English because she was fond of her former English high school teacher. Her specific aim for the intensive reading course was to polish up her speaking, reading, and English grammar skills, which corresponds with the self-evaluation of her four skills, as she ranked her speaking and reading to be weaker than writing and listening.

In Meng’s first reflective journal, she considered her performance in that session to be a “failure” [Meng CJ1]. She explained that she had not previewed the lesson and was afraid of being called upon by the teacher and not understanding his question. In the interview, she stated that she would preview the lesson before the next session, and she made up her mind to “speak up at least once in that session.” It suggests that her frustration could motivate her toward positive change.

She also explained that her disengagement in other cases was because she tended to overthink and thus missed opportunities to speak up. As she had it, “I hesitated and pondered over every word” [Meng CI1, L31]. However, in her second reflective journal Meng admitted she “enjoyed” the lesson when she “dared to answer a question voluntarily.” Her participative role changed from a passive listener to an active speaker, which resulted in her emotional experience moving from negative to positive.

In the following interactional event, which occurred during the session after the second interview, Meng seemed to be more active than in the previous session as she voluntarily participated three times. She also appeared to be more confident. The illustrative case featured the second interactional event involving Meng as a speaker. In this session, the teacher guided the students through the article *Soldier’s heart*. Unlike in the traditional teacher-centered model, the teacher asked each student to read one paragraph aloud and then asked a question or provided some comments. The teacher explained that this activity aims to encourage students’ engagement in interaction and create an inviting atmosphere for interaction.

In the first interactional event in that session (“remember the war history”), when the students discussed why young people “cannot remember” war history, Meng argued that since ordinary people never experienced war, they might be indifferent to it [Class CK5, L165]. She disagreed with other students’ ideas by saying, “I did not agree with you totally” [Class CK5, L154]. However, what she meant to say, as she explained in the interview, was, “I disagree with you partially” [Meng CI3, L2]. The teacher was confused, but he realized that she intended to express a partial negation. Meng confirmed that she was “a little embarrassed” due to his misunderstanding [Meng CJ3, L3]. However, it did not affect her later participation in that session. She appeared to be resilient, which may stem from her prior experience of overcoming adverse situations.

The second interactional event that Meng participated in was a detailed discussion of paragraph 22 in the same article. They primarily focused on one sentence: “The men and women I worked with in universities were pale and unreal in comparison.” One student asked why the word “pale” was used here. Meng was involved in this discussion.

### Observer’s Perspective

The following excerpt is the transcript of the classroom recording showing the interaction between the teacher and the student Meng who asked for clarification.

**Excerpt 1:**
236 T: … “The men and women I worked with in universities were pale in comparison.” So ‘the university teachers and staff they were pale” =
237 S1: = Why “pale”?
238 T: They were like, ok. Why pale? Good question. When somebody is weak, when somebody has just recovered from illness, you might say this person looks pale. Pale means powerless at least or weak. So the people in the university are weak and powerless, and also unreal. They are fake. Ok? [C]-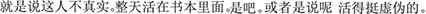-[C] (That is to say someone is unreal like living in a world of books. Right? Or, live hypocritically.) I’m not sure if I am one of them.
239 Class: No. $$ (laughter)
240 T: Maybe I am becoming one. Maybe I am becoming one. “They were hollow and filled with words.” (Meng puts up her hand.) Yes, please. (Meng stands up)
241 Meng: From this paragraph, I think experience is, err, very important. Take our class as an example.
242 T: Uh-huh.
243 Meng: Listening to our teachers can give us a shallow understanding of this article, but engage us in this class by talking about our ideas, can make a better understanding.
244 T: Ok.
245 Meng: Err, if we experience the war and …
246 Ss: $$ (laughter)
247 T: $ I can engage you in a war if you like.
248 Ss: $$ (laughter)
249 Meng: We will, we will, maybe we will understand things better than the author.
251 Ss: $$ (laughter) Ah. Yes.
252 T: Are you following me? Do you know what I am talking about?
253 Ss: Yes.
254 Ss: $$ (laughter)
255 T + Ss 1: Tell me I forget, show me I remember, involve me, I learn. 2: show me I remember, involve me, I learn.
256 T: Ok. I think this is a good way because when I ask you, you can have any questions or comments. I share your understanding, and also I know where your puzzles are. Ok? And it feels like we are learning this one together. Ok. And I am still filled with words, but I am not “hollow”$.
[Class CK5, L236-259]

The teacher welcomed the student’s question and reacted positively, saying “good question” (line 238). Then he went on to explain the meaning of the word and elaborated on its use in line with the author’s opinion: “they were weak and powerless and also unreal’ (line 238).” After that, the teacher switched to Chinese, ensuring his students understood the more profound message behind the article.

In what followed, the teacher started to mock himself, saying, “I am not sure if I am one of them…Maybe I am becoming one.” This utterance made the whole class laugh. The laughter throughout this excerpt indicates the enjoyment and positive emotion in this interaction. It seems that the teacher’s self-revelation and mockery lightened the mood and drew all participants closer. This inviting atmosphere also encouraged Meng to take part in the interaction. In line 245, Meng compares war experiences to learning in a classroom to explain why the people in the article who had no first-hand experience of war felt hollow. The teacher replied by making a joke: “I can engage you in war if you like” (line 247). His remark lightened up the atmosphere and made the whole class laugh (line 248). Though not directly linked to the intended pedagogical aim, the joke attends to students’ emotions and shortens the distance between students and teachers. The teacher summarized Meng’s idea and extended it to a famous Chinese saying. The repetition of the quote in the chorus also suggests a harmonious relationship between the student and the teacher. The phrase “Tell me, I forget; show me, I remember, involve me, I learn” is essential for understanding the process of interaction and the role of positive emotions in fostering learner engagement.

The above observational data reveal the participants’ positive emotions in the interactional event. The following section presents students’ perspectives of this interaction based on corresponding reflective data.

### Students’ Perspectives

Meng’s emotions in this interactional event can be revealed in her reflective journal and interview. She also reflects on her shifting attitudes and the influence of that shift on her participation. Most strikingly, Meng came to the researcher after the session and said excitedly, “can you interview me now? I have so much to say.” She could not wait to share the positive emotions she had experienced.

**Excerpt 2:**
In this process, the teacher nodded and said “hmm” whenever I finished one sentence (laughter). I noticed when I participated in the interaction for the second time and mentioned, “be engaged in our class,” the teacher said, “Ok. Good.” I was delighted. The teacher changed a word, which showed that he agreed with my opinion. I might have said something that he also wanted to say, so I was thrilled at that time. Then when I was about to say my third point, the teacher interrupted me and said something else. The teacher thought of something that could boost the classroom atmosphere. All the students laughed because of the teacher’s sentence. Students who did not pay much attention to my response would listen to me carefully after that.
[Meng CI3, L62]

Meng expresses positive emotions (“delighted,” line 3) when the teacher agrees with her opinion by nodding and saying encouraging words. She appreciates the teacher’s humorous remark as an effective means of creating a vibrant, positive atmosphere for interaction. Meng believes that the teacher stimulated positive emotions and created a relaxing atmosphere that could positively affect students’ active involvement (lines 7–8). Her excitement after this session formed a considerable contrast with the previous disengagement in the previous class meeting. Her previous negative emotions, such as “nervousness,” “shame,” “frustration” [Meng CJ1], motivate her to participate actively in a later session. However, those negative emotions changed into positive ones upon receiving the positive appraisal from the teacher. She was “thrilled” and “delighted,” which testifies to the teacher’s significant influence on students’ engagement and emotion in interaction. In the interview, she also comments on her emotional change when comparing her two involvements in the same session.

**Excerpt 3:**
I thought I was ridiculous in the previous instance (laughter), but the second time was relatively fluent. Although it seems just ok when I listen to it again now, however, compared with the previous one, this time is a bit better. So when I spoke for the second time, I was not as nervous as the first time. This time, I made my point clearer and expressed what I wanted to say, although I did not use complicated words as I thought $ (laughter). I used a lot of simple words. When I sat down, I did not notice that I made any grammatical mistakes. So I was pretty satisfied.

[Meng CI3, L69]

She first reflects on her performance in the first involvement, in which she made a grammatical mistake. She intends to express partial agreement, but she uses the phrase “totally disagree,” a mistake which she explains in the journal is due to her extreme nervousness. However, in the second instance, she reports being more relaxed and confident by consciously monitoring and evaluating the accuracy of her answer while taking the seat. She explains that she used to be obsessed with avoiding making grammatical mistakes, which affected her speaking fluency. This time, she was satisfied with her performance as she made no obvious grammatical mistakes, which shows her level of negative emotions in interaction has dropped, while her positive emotions became prominent.

Explaining the effect of this emotional change, Meng said that the positive emotion helped her perform better in the second round. She was no longer discouraged and embarrassed. On the contrary, she continued to participate actively with excitement and enjoyment. She even voiced her opinion for the third time in a 45-min session, which is rare in large class size with more than 20 students. When Meng spoke for the third time, the teacher asked her a follow-up question. Instead of answering the question, she threw the question back to the teacher: “What is your opinion?” [CK5, L319]. She explained that she could not express herself clearly, so she sought the teacher’s help. Her response reveals the relaxed atmosphere and rapport between the student and teacher. In the traditional teaching model based on Confucian values, students are expected to respect the teacher and seldom challenge them; they also apologize to the teacher if they fail to answer a question.

Nevertheless, in this case, the teacher was not offended. Instead, he accepted the challenge. The teacher’s perspective on this incident will be presented in the next section.

The satisfaction and confidence obtained in the previous interactions motivated Meng to engage in the session actively. Her positive emotions might also result from adopting the “student’s question and comment” interaction pattern. As Meng reflected, “the more I participated verbally, the more confident I would become” [Meng CI3, L8]. Importantly, this positive learning experience might also impact her future class participation and enhance her self-esteem. As Meng admitted, “When I was excited to the point that the excitement overcame the nervousness, I will say it better” [Meng CI3, L107]. Clearly, positive emotions surpass negative emotions in the learning context and prompt better performance in classroom interaction. This resonates with a balanced interactive model of PP 2.0, which may involve transcendence in the adaptive process toward growth and development (Wong, 2011).

Another student, Piao, who was involved in this interaction as a hearer, also stressed the role of positive emotions in fostering engagement. However, unlike Meng she did not experience any strong negative or positive emotions. Similarly, instead of hindering participation, negative emotions could promote participation if the motivation is strong.

**Excerpt 4:**
Maybe other students have the same thoughts because we are too shy and eager to reach perfection. When we were in senior high school, our teachers would ask us to answer questions in class. Most of us are familiar with this teaching method, so we still wait for the teachers’ asking in university. However, our teaching method is different from the past, so we should try to gain the chances ourselves. Our hard work is the foundation of our courage. If we want to express our feelings and have good communication with our teachers, we must prepare before class. Thus, I think all of us should work hard and overcome ourselves at first.
[Piao CJ3]

In Piao’s reflective journal entry, she explains the traditional teacher-led question and answer pattern of interaction deeply rooted in the English language classrooms in China. Negative emotions are associated with the traditional teaching method, suggesting that students’ emotions are also culturally sensitive. Having been accustomed to the traditional model, which was prominent in high schools, Piao senses the need to adapt to the new way her university teacher promotes. She describes the complexity of students’ emotional experience, which involves negative emotions, such as tension due to shyness and striving for impeccable performance at the same time. There is a cultural aspect to this emotional turmoil, as the students want to provide correct answers and avoid mistakes for fear of losing face.

Meanwhile, as Piao points out, students consider active participation as “chances to gain by ourselves” and are eager to communicate with the teacher. This shows that their awareness transcends negative emotions: they believe in positive results from active participation. In order to overcome her shyness, the student believes that preparation and hard work could equip her with courage for active participation, which is similar to Meng’s view, which makes it her priority to participate actively at least once in a session. It shows that the tensions at the interpersonal level may lead to a change of emotions and attitude toward class participation.

Students’ emotions fluctuated as they performed and switched their participative roles. Students who volunteered to participate in an interaction expressed more positive emotions than negatives. Meng expressed higher levels of positive emotions as a volunteer student, which stood in stark contrast to her negative experiences in the previous session. Other students who participated voluntarily also reported to have “enjoyed” the classroom interaction. For instance, Tian said she felt better prepared and found it “less threatening” [Tian I1, L87]. Peng felt “fantastic” when the teacher asked her to explain a word in English, and she gave a correct answer, which left a deep impression a few days after the occurrence [Peng I3, L16]. Their positive emotions resulting from active engagement in class discussions may also include the sense of achievement upon hearing the teacher’s approval.

Students who were called to answer expressed negative emotional experiences. In one such case, Lan was “nervous” because her “spoken English was not very good,” and she was afraid of making mistakes [LanJ1, L2]. When she was called on, she was timid due to “lack of confidence” [LuI1, L4]. Meng explained she was afraid of being laughed at by others and losing face [MengI1, L8].

Students involved in the interaction as hearers may also feel stressed and frustrated when they witness their peers’ active participation. As Jun reported, “They put up hands all the time, and they made me stressed” [JunI1, L101]. Highly motivated students sensed that they were losing opportunities. They either “envied” others’ comments [PiaoJ3] or “admired” someone who received the teacher’s positive feedback [Zhao I2, L16].

The students’ responses demonstrate that learners’ emotions are influenced by their own perceptions, others’ reactions and cultural expectations. For example, some students were “thrilled when the teacher showed acceptance to their answers” [Zhi I2, L140]. They sensed that “teachers favor those active students in the class” [Tian I1, L89]. They also reflected that they “gained confidence” and would be willing to participate in similar activities in the future [Lan I3, L44].

The pattern of interaction also affected their emotional experience. The students described the teacher’s attempt to encourage “students’ questions and comments” as “interesting” and also “useful” for their learning [Piao J2]. Their observation suggests that their positive emotions are stimulated by teachers’ attempts in arousing their interest, especially allowing students to take the initiative in interaction. The students did appreciate the various attempts that the teacher made to make his class more engaging. They found the new pattern more challenging than traditional teacher-led interaction, but they also understood its learning benefits.

Students’ perspective illustrates the role of positive emotions and the complex interplay of positive and negative emotions in language classroom learning, which adds to the simple observation of what is going on as perceived by researchers. In addition, the teacher’s perspective is also equally important in understanding what is going on in the classroom.

### Teachers’ Perspective

As for the teacher, he was aware of the importance of promoting students’ engagement by avoiding tasks and actions that could arouse students’ negative emotions. As he explains, the purpose of the new interaction pattern was to keep students’ emotional needs in mind.

**Excerpt 5:**
“Students’ questions and comments” is a kind of active interaction. Let every student feel that he or she is valued in the classroom, so students will be enthused to learn. Otherwise, the traditional way may make interaction fall into a trap: the teacher tends to ask a few active students, which only accounts for a quarter of the students at most. Then you repeatedly ask them to answer your questions. It is a good cycle for these students in the longer term, but it will be a downward spiral for other students. Those inactive students may think they are already like this and they will not cooperate with you. Interaction should be with the whole class. The teacher should let them feel the class is very democratic. Like I said, if you don’t know, you can ask a question. At least, he has something to say. If he/she knows, then he/she can explain that idea and show it to other classmates. I try to make sure every student has a distinct voice or discourse power.
[TCI3, L39–42]

The teacher regards this pattern as an “active interaction” that aims to stimulate students’ positive emotions by providing more opportunities and flexible choices or a certain degree of freedom for students. He intends to avoid the “downward spiral.” Inactive students remain inactive and unmotivated or even keep silent, all of which trigger negative emotions. It is often the case if students do not feel equally “valued.” Although such students may not be judged as “inactive” in a traditional Chinese classroom, they may not be willing to participate. Their negative emotions may persist, which may prevent them from making any progress. The teacher also stresses the need for students of different proficiency levels to participate. He believes that the broader coverage of the students could create a more “democratic” classroom. This excerpt shows that the teacher paid particular attention to students’ emotional experience in interaction. He also intended to create a relaxing and friendly environment for promoting positive emotions for classroom interaction, which the ongoing English educational reform might influence in China and the influence of communicative language teachers.

The teacher also reflects on Meng’s performance, especially when Meng challenged him and threw the question back at him.

**Excerpt 6:**
When I asked her, she said she didn’t know. Then she said, “teacher, what is your opinion?” Such, I would say…This behavior was based on the premise that she was pretty familiar with the teacher and was quite democratic. Given a relatively stern teacher, if the teacher asked you a question and you don’t know but threw it back to him, the teacher would be angry. However, I get along well with my students, so I don’t mind her saying, “I don’t know. Help me answer it.” It’s not a problem.
[TCI3, L60]

The teacher explains how a traditional Chinese teacher would behave instead of how he and his student acted. From a traditional Chinese point of view, teachers are highly respected and disobedient students need to be disciplined. A more conservative teacher would be angry with Meng. However, the teacher in the study positions himself as a more “westernized” teacher who advocates democracy in classrooms and uses humor to create positive emotions. In this excerpt, the teacher is pleased to accept the challenge and explain rather than take offense. He interprets the student’s behavior as caused by the friendly student-teacher relationship and a relaxing atmosphere.

On the other hand, the teacher’s reflective data also shows that he is aware of protecting students’ faces and ensuring they feel at ease in the classroom. He worries that the students’ positive emotions might change into negative ones if he gives corrective feedback or negative evaluation. As the teacher explained in another interview, “If something is wrong with his answer, while he is trying to express something, I normally do not interrupt him because I fear that correction may affect his enthusiasm.” He also commented on the relaxed atmosphere of that session, which to some extent, explained why Meng behaved so actively.

**Excerpt 7:**
They thought they were more relaxed and in a good state that day. The interactional atmosphere was sometimes like a competition. It might be just several students who were in a good state. Then, they competed to have the floor before others. Other students were also affected and did not want to keep silent anymore. They all want to stand up and say something. This situation has happened before.
[TCI3, L53]

The teacher seems satisfied with the atmosphere and the students’ overall performance. He also explains why students’ positive emotions led to their active participation. As he believes, they “competed to have the floor,” which vividly reveals their emotional state and the interaction atmosphere as perceived by the teacher. The teacher observes that this collective positive emotion was usually stimulated by “several active students.” Their active engagement would influence other classmates. Even students who were usually reluctant to participate would also be affected and thus persuaded into more active involvement. It shows that the emotions of an individual student change in interaction. Their emotions are socially constructed during student-teacher interaction. Students’ emotions may be affected by their teacher, their peers, and the ongoing interaction.

## Discussion and Implications

### The Nature of Students’ Emotional Experience in Student-Teacher Interaction

The study found that student-teacher interaction affected the students’ emotional responses in EFL classrooms in different ways. To be more specific, various levels of enjoyment and anxiety were revealed when the students were involved in different participant roles and patterns of interaction and when they held different self-perceptions of their performance.

First, students’ dynamic participative roles in interaction affected their emotions. A volunteer student like Meng intends to experience a more intense level of enjoyment after her active participation in class. She expressed confidence and was excited as a result of her engagement. The students whom the teacher called to answer, experienced negative emotions, such as stress or fear. However, the teacher’s positive feedback or ending of student-teacher interaction could ease their negative emotions to some extent. The students also reported that their emotions changed after classroom interaction, which usually involved fear before the session and possible enjoyment and satisfaction after the interaction.

Students who acted as hearers or just as the audience in classroom interaction may also experience negative emotions because they may experience frustration for losing learning opportunities. Secondly, patterns of student-teacher interaction affected students’ emotions. Some participants reported more negative emotions concerning the teacher-led interaction, which is common in this educational context. The teacher’s effort to introduce a more student-centered interaction pattern, such as “students’ questions and comments,” resulted in lowering students’ negative emotions. Thirdly, students’ positive and negative emotions were affected by their evaluation of their performance and their language proficiency. If they hold a low self-evaluation or perform less satisfactorily, they tend to be more anxious. This confirms previous findings on the correlation between academic enjoyment and self-perceptions (Goetz et al., 2008).

Thus, the nature of students’ emotional experience in interaction is complex and dynamic. This finding is consistent with Boudreau et al. (2018) ‘s study, which explores individual learners’ moment-by-moment enjoyment and anxiety during a communication task. Instead of treating learners’ emotions as stable individual difference variables, their positive and negative emotions are highly dynamic.

Informed by PP 2.0 in SLA, MacIntyre et al. (2019) promote the idea that students’ emotional experiences to some extent depend on the social and cultural context. This observation also holds true for our study. Most of the time, the nervousness described by students is associated with the need to save face, which is vital in the Chinese cultural context. Thus, the desire to provide a correct answer and the need to save face sometimes may cause tensions. However, more positive emotions can emerge with the recent influence of the communicative approach, or the “western” model, as recognized by students and teachers. Teachers are increasingly aware of the importance of developing students’ communicative competence as informed by the national curriculum, and in fact, they prefer a dialogic and democratic interaction. Students in different cultural and educational contexts may bring different emotions to their classroom interaction. This study thus supports Jiang and Dewaele (2019) on the uniqueness of Chinese foreign language learners’ classroom emotions. Chinese students tend to be more anxious than students from other countries, which might be related to the tradition of test-centered education. Emotion is a social being, which is shaped by the sociocultural and educational context.

Researchers should focus on the weaknesses, strengths, and resources these diverse learners bring to interaction and learning. Teachers and researchers also need to be sensitive to the participants’ past experiences and cultural values in understanding their emotional experience in interaction for language learning.

### The Interplay of Positive and Negative Emotions in EFL Classroom Interaction

As for the interplay of positive and negative emotions in interaction, the study found that a certain level of negative emotions may lead to a higher level of positive emotions. As in Meng’s case, her previous frustration and anxiety caused by her unsatisfactory performance heightened her later excitement and enjoyment. This finding confirms PP 2.0 ’s view on the balanced interactive model of positive and negative emotions (Wong, 2011). It also answers MacIntyre et al.’s (2019) call to draw the field’s attention to acknowledging the interaction between positive and negative phenomena. A simplistic distinction on positive and negative emotions as “good or bad” would not be enough, as learners’ emotional experiences are far more complex.

It suggests no one-to-one relationship in which positive emotions lead to learning, and negative emotions hinder interaction. Students’ positive emotions were usually expressed when students reflected their emotions after engaging in interaction. The reflective data suggests that students’ positive emotions stimulate verbal exchanges with others. When it comes to negative emotions, teachers try to avoid them as hindrances to active participation. However, students’ reflective data suggests that they can both inhibit and promote interaction. Students’ negative emotions that emerge in interaction do not always prevent students from participating in-class activities. On the contrary, when the motivation is strong enough, based on students’ self-perception, motivation, and interpretation of the interaction situation, the negative emotion can stimulate students’ active participation. As MacIntyre and Gregersen (2012) point out, the interactions among positive and negative emotions could stimulate learning in different ways.

Considering positive and negative emotions and anything in-between, a broader spectrum of emotions can offer a more holistic understanding of students’ emotional experiences. What is more, how students’ negative emotions transfer or transcend into positive emotions is more important in this quest, harnessing the possible benefits of negative emotions for individual learners. As the study suggests, students’ emotional experience, despite individual differences, may be influenced by the change of interactional pattern and the teacher’s feedback. Therefore, teachers need to provide encouragement and constructive feedback and create enough space for interaction and reflection for emotions which is beneficial for classroom engagement and learning.

### Understanding Emotional Experience From Multiple Perspectives

The triangulation of this study made it possible to understand different perspectives on emotional experiences. We were, to some extent, able to identify both students’ overt and covert emotional experiences based on the observational data. As discussed in the previous section, the Chinese educational context affected the management of their emotions. A given student may always have private, hidden internal emotions as well as publicly displayed emotions. Therefore, observational data could only provide a partial picture. From students’ and teachers’ reflective data, more positive and negative emotions and changes of emotion can be revealed. In addition, students and teachers were also constantly interpreting and evaluating each other’s feelings. Students tried to read their teacher’s emotions and decide on their subsequent interactions. This finding supports Swain (2013)’s argument on the interpersonal nature of emotions. In the L2 learning process, emotions are socially and culturally co-constructed together with cognition, which means cognition and emotions are interdependent and inseparable. Whether acting as speakers or hearers, students were determined to please teachers and keep their expectations in mind. Thus, their emotional experiences were also mutually informing and co-constructed, which is based on mutual interpretation. Their dynamic experiences are embedded in this interpersonal exchange.

It can also be observed that the teacher in our study aimed to promote students’ positive emotions and eliminate possible negative emotions, which they believed might hinder interaction. This finding is consistent with previous survey studies on various internal and external factors affecting FLE and FLCA (Li et al., 2018; Jiang and Dewaele, 2019). Teachers and peers play a vital role in shaping students’ experiences. The present study’s design enables students’ emotional experience in moment-to-moment natural classroom interaction to be revealed.

Most of the students’ negative emotions were identified through reflective data. Compared with the positive emotions, which can be observed, most negative emotions were expressed in the reflective data. It is especially in their reflective journals that students took account of their emotions, change of emotions, and its influence on participation. Students’ positive emotions may also emerge from the chance to provide self-reflection after classroom activities.

Therefore, the study suggests that researchers need to elicit multiple data sets that provide different perspectives to understand students’ emotional experiences. For example, the design of this study enables eliciting the views from the observer, teachers, students in speakers’ and hearers’ roles. Multiple perspectives may converge, but their mutual interpretation may also reveal mismatches (Dewaele and MacIntyre, 2016). found that what learners reported as enjoyable activities were considered risky for teachers due to potential misinterpretations.

In addition, students’ multiple interviews conducted over a certain period were collected in the three cycles of data analysis, which provides chances for changes to appear. When viewed as a metatheory, this study has confirmed that PP enables and encourages SLA researchers to adopt a wide range of approaches (MacIntyre et al., 2019), which provide situated evidence-based answers to classroom-related questions. These quantitative, qualitative, or mixed methods will stimulate rich findings from diverse contexts.

As for the pedagogical value, the findings of this study have implications for foreign language learning, teaching, and teacher training. First of all, given the dynamic nature of students’ emotions, teachers and teacher trainers need to promote a positive classroom ecology by using humor or adopting different interactive patterns and activities. The study found that some negative emotions, such as stress or anxiety, may also be changed into positive emotions through interaction. Teachers need to be aware of students’ emotional changes in interaction. They should also encourage students to involve in meaningful interactions. Secondly, teachers should guide students to better prepare for classroom content and emotions, inviting them to reflect on their learning outcomes. It has been observed that students’ better preparation before class may lead to more confidence, and the sense of achievement from previous experiences may lead to positive emotions.

## Conclusion and Limitations

Positive psychology 2.0 in SLA offers a balanced model of investigating the interplay between positive and negative emotions. The triangulation of the present study enables individual students’ observable and unobservable emotions to be analyzed around classroom interactions within specific immediate, cultural and educational contexts. By analyzing a representative interactional event and looking across interactional events, we explored how individual students’ emotions fluctuate and how positive and negative emotions interact and stimulate different active tendencies.

Based on the previous data analysis and discussion, implications were derived from students’ and teachers’ understanding of emotional experience in interaction. First, students’ emotional experience in EFL classroom interaction is complex, dynamic, and context-sensitive. Learners constantly interpret others’ emotions and manage their feelings, reflecting the con-constructive nature of emotional experience. Secondly, to capture its complexity, the relationship between positive and negative emotions needs to be understood in terms of a specific individual, immediate and educational contexts. The focus either on positive or negative emotions alone fails to provide a clear picture. An understanding of positive psychology in learning needs to include the transformation of negative emotions. Thirdly, students’ emotional experience needs to be discussed from different perspectives, which means that observational and reflective views can reflect different dimensions of their emotional experience. Students’ roles and the immediate context also need to be considered when we look at students’ experiences. The “interactional event” construct provides one way of extending researchers’ and English teachers’ current understanding of students’ emotional experience in interaction holistically.

This study responds to the call (MacIntyre et al., 2019) to apply the second wave of positive psychology principles in SLA to view learners’ emotional experience in classroom interaction comprehensively. It shows the dynamic and complexity of students’ shifting emotions as they participate in class discussions over 2 months. The study suggests that students’ positive emotions could transcend negative emotions and influence their engagement in classroom interaction. It is vital to integrate positive and negative emotions promoted by PP2 to understand how emotional experience influences interaction and classroom language learning. What needs to be avoided is a static view of students’ emotions in interaction. In addition, teachers and researchers need to respect and recognize the importance of changing contexts.

This study has some limitations. First, its scope is restricted to one location in China; thus, it only accounts for the experience of interaction in one particular context. Secondly, though the construct of “interactional events” is helpful to explore the experience of interaction in a more holistic way, its boundaries might be subject to different researchers’ understanding. Thirdly, gender may be a key factor influencing students’ experience in interaction, but this issue has not been addressed here.

Though this study is limited to the Chinese context, its findings may apply to EFL classrooms in other Asian countries. It will be noteworthy to adapt the construct of interactional events and the design of this study to investigate students’ emotional experiences in another educational context. As for future research directions, the discussion of emotional experience in interaction may focus on the emotions that emerge in interaction and the benefits of positive emotions for learning through interaction.

## Data Availability Statement

The original contributions presented in the study are included in the article/supplementary material, further inquiries can be directed to the corresponding author.

## Ethics Statement

The studies involving human participants were reviewed and approved by University of South Australia. The patients/participants provided their written informed consent to participate in this study.

## Author Contributions

YW conceived and designed the study, collected and analyzed the data, and drafted the first manuscript. MM revised the manuscript. Both authors contributed to the article and approved the submitted version.

## Conflict of Interest

The authors declare that the research was conducted in the absence of any commercial or financial relationships that could be construed as a potential conflict of interest.

## Publisher’s Note

All claims expressed in this article are solely those of the authors and do not necessarily represent those of their affiliated organizations, or those of the publisher, the editors and the reviewers. Any product that may be evaluated in this article, or claim that may be made by its manufacturer, is not guaranteed or endorsed by the publisher.
